# Time-Resolved Information-Theoretic and Spectral Analysis of fNIRS Signals from Multi-Channel Prototypal Device

**DOI:** 10.3390/e27070694

**Published:** 2025-06-28

**Authors:** Irene Franzone, Yuri Antonacci, Fabrizio Giuliano, Riccardo Pernice, Alessandro Busacca, Luca Faes, Giuseppe Costantino Giaconia

**Affiliations:** 1 Department of Engineering, University of Palermo, 90128 Palermo, Italy; irene.franzone@unipa.it (I.F.); fabrizio.giuliano@unipa.it (F.G.); riccardo.pernice@unipa.it (R.P.); alessandro.busacca@unipa.it (A.B.); luca.faes@unipa.it (L.F.); costantino.giaconia@unipa.it (G.C.G.); 2 Faculty of Technical Sciences, University of Novi Sad, Trg Dositeja Obradovića 6, 21102 Novi Sad, Serbia

**Keywords:** network physiology, functional near-infrared spectroscopy, power spectral density, information dynamics, time-resolved analysis, time-varying autoregressive modeling, recursive least-squares

## Abstract

Functional near-infrared spectroscopy (fNIRS) is a non-invasive imaging technique that measures brain hemodynamic activity by detecting changes in oxyhemoglobin and deoxyhemoglobin concentrations using light in the near-infrared spectrum. This study aims to provide a comprehensive characterization of fNIRS signals acquired with a prototypal continuous-wave fNIRS device during a breath-holding task, to evaluate the impact of respiratory activity on scalp hemodynamics within the framework of Network Physiology. To this end, information-theoretic and spectral analysis methods were applied to characterize the dynamics of fNIRS signals. In the time domain, time-resolved information-theoretic measures, including entropy, conditional entropy and, information storage, were employed to assess the complexity and predictability of the fNIRS signals. These measures highlighted distinct informational dynamics across the breathing and apnea phases, with conditional entropy showing a significant modulation driven by respiratory activity. In the frequency domain, power spectral density was estimated using a parametric method, allowing the identification of distinct frequency bands related to vascular and respiratory components. The analysis revealed significant modulations in both the amplitude and frequency of oscillations during the task, particularly in the high-frequency band associated with respiratory activity. Our observations demonstrate that the proposed analysis provides novel insights into the characterization of fNIRS signals, enhancing the understanding of the impact of task-induced peripheral cardiovascular responses on NIRS hemodynamics.

## 1. Introduction

Functional near-infrared spectroscopy (fNIRS) is a non-invasive imaging technique which relies on the use of light at specific wavelengths for measuring the hemodynamic activity of brain tissue [1]. Specifically, the near-infrared region (650–950 nm) enables the estimation of the concentration of oxyhemoglobin $\left(HbO_{2}\right)$ and deoxyhemoglobin (HHb) by exploiting their different absorption spectra at different wavelengths [2,3]. Among the different fNIRS techniques available on the market, the continuous-wave (CW) modality employs constant tissue illumination to quantify light attenuation through the head. Given its reliance on low-cost electronic components, as well as its high portability and ease of use, the CW modality has become the most commonly used technique. Indeed, only recently novel multichannel fNIRS systems were developed to answer the need of a portable system that can monitor hemodynamic activity in ecological and clinical settings [4,5].

In a recent study, an integrated system was developed that combines a novel continuous-wave fNIRS device with a modified commercial electroencephalographic (EEG) system [4]. This hybrid system has been validated for monitoring Event-Related Potentials across three distinct experimental paradigms designed to elicit the following: (i) motor cortex activity via a finger-tapping task [6]; (ii) visual cortex activity using a flickering black-and-white checkerboard at a frequency of 2 Hz [7]; and (iii) frontal cortex activity through a Stroop task [8].

However, it is well established that cognitive and emotional tasks can influence peripheral physiology by altering heart rate, respiration, blood pressure, and skin conductance [9,10]. These physiological changes affect both scalp and brain hemodynamics, thereby contributing to the fNIRS signal in ways that extend beyond neural activity [11]. In particular, systemic physiological variations—especially those driven by autonomic or cardiorespiratory activity—can impact both superficial and intracerebral hemodynamics. Previous research has shown that signals recorded from a given scalp location do not exclusively reflect neurovascular coupling but may also result from hemodynamic changes in extracerebral tissues (e.g., skin, muscles, skull) as well as in deeper brain regions influenced by systemic processes [12]. This issue is especially pronounced in CW fNIRS systems with short source–detector separations (typically < 4 cm), where the sensitivity to extracerebral tissues often exceeds that to cortical areas. Recent studies confirm that the extracerebral compartment—including scalp, skull, and cerebrospinal fluid—absorbs a substantial portion of the incident light, often leading to signals dominated by superficial layers [13,14,15]. This limited depth sensitivity remains a major limitation of CW fNIRS, particularly in adult populations, and must be considered when interpreting signal origin.

As a result, fNIRS measurements are affected by various background physiological components that are often considered “noise” in traditional analyses of brain connectivity, as they can obscure the detection of evoked neural responses. Yet, the influence of these systemic signals on both brain and scalp hemodynamics remains underexplored. Most analysis techniques aim to remove such components from the fNIRS signal [16,17]. Recent developments in the field of network physiology, however, propose a paradigm shift: rather than viewing these fluctuations as artifacts, they can be interpreted as meaningful indicators of physiological interactions [18,19]. According to this perspective, the human body can be seen as an integrated network, where each physiological system, despite having its own regulatory mechanisms, continuously interacts with others to coordinate functions and generate distinct physiological states in both health and disease [20]. In this framework, cardiorespiratory dynamics arise from the coordination of respiratory, cardiovagal, and sympathetic functions, which operate across various levels of the nervous system. These interactions play a critical role in maintaining physiological equilibrium and have only recently been shown to have a direct impact on both scalp and neural signals in specific clinical conditions [21,22]. To investigate these complex interactions, we designed a protocol based on the breath-holding task, in which the presence of respiratory signals is systematically modulated, allowing for a detailed assessment of the influence of respiratory activity on the local concentration of oxy- and deoxyhemoglobin measured over the scalp [23]. While previous studies have demonstrated strong responses in all brain chromophores due to respiratory activity using well-established methods [17], traditional approaches do not fully characterize the regularity, complexity, or transient dynamics of these signals.

Therefore, the aim of this work is to characterize the effects of respiratory activity on scalp hemodynamics as measured by a prototypal fNIRS system. We employ a multi-domain analysis that includes state-of-the-art signal processing techniques, which have not yet been extensively applied in the context of fNIRS signal analysis. These techniques are designed to assess physiological oscillations and their modulation during breath-holding, as well as the transient dynamics during the intermittent presence of respiratory activity. Specifically, to investigate the complexity and predictability of the fNIRS signals dynamics, we leverage information-theoretic measures computed in a time-resolved fashion such as conditional entropy (CE) [24] and information storage (IS) [25]. Moreover, given the presence of oscillatory activity that can be measured over the scalp, we perform a frequency-domain analysis to explore the distribution of power across frequency bands, providing an indirect measure of both the intensity and temporal evolution of neural activity [26].

## 2. Materials and Methods

### 2.1. Dataset Description and Pre-Processing

A total of 6 healthy subjects (4 males, 2 females; mean age: 27 ± 2.5 years) were enrolled in this study and provided informed consent prior to participation. The experimental procedure was approved by the ethical committee of the University of Palermo and was conducted in accordance with the ethical standards of the Helsinki Declaration. The subjects were seated in a chair in an isolated room and instructed to breathe normally, avoiding other actions or excessive movements. The experimental protocol consisted of a five-minute recording alternating two different phases: (i) apnea episodes, during which subjects were instructed to hold their breath, starting at predefined time points (8, 50, 110, 170, and 230 s), and (ii) a breathing phase, during which the subject resumed normal breathing before the onset of the subsequent apnea.

**Figure 1:**
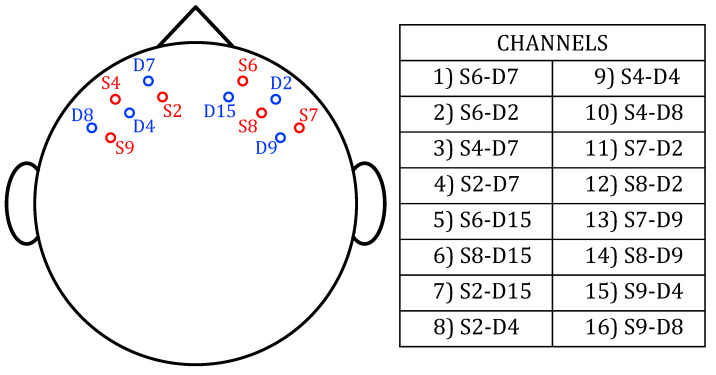
fNIRS optodes configuration and selected channels.

The signal acquisitions were performed using a multi-channel CW-fNIRS system. This system includes 16 bicolor light-emitting diodes (LEDs) operating at wavelengths of 735 nm and 850 nm, alongside 16 silicon photomultipliers (SiPMs). Each SiPM is positioned approximately 3 cm away from its corresponding LED source and acquires signals at a sampling frequency ($f_{s}$) of 130 Hz. This is in line with previous studies with a similar setup [22,27], and with a work demonstrating that, under optimal conditions, optode spacings up to 5 cm are usable with NIRS equipment [28]. According to a “banana-shaped” model, which assumes that the penetration is similar for all wavelengths, the resulting wavepath had a maximum penetration depth of about 1.5 cm [29,30]. Thus, these fNIRS measurements are a superposition of signals from multiple tissue layers, with dominant contributions from extracerebral tissues [14,15]. Further details about the prototype electronic components and the realization procedure can be found in [4]. The selected configuration of the optodes is shown in Figure 1, where each optode is labeled with the letter ‘S’ (source) if it is an LED, or ‘D’ (detector) if it is a SiPM. Each optode is also assigned a number to identify its position on the scalp. In particular, the positions of the 6 LEDs are marked in red, while the 6 SiPMs are marked in blue. Together, they provide 16 different channels, which are listed in the table of the same figure.

**Figure 2:**
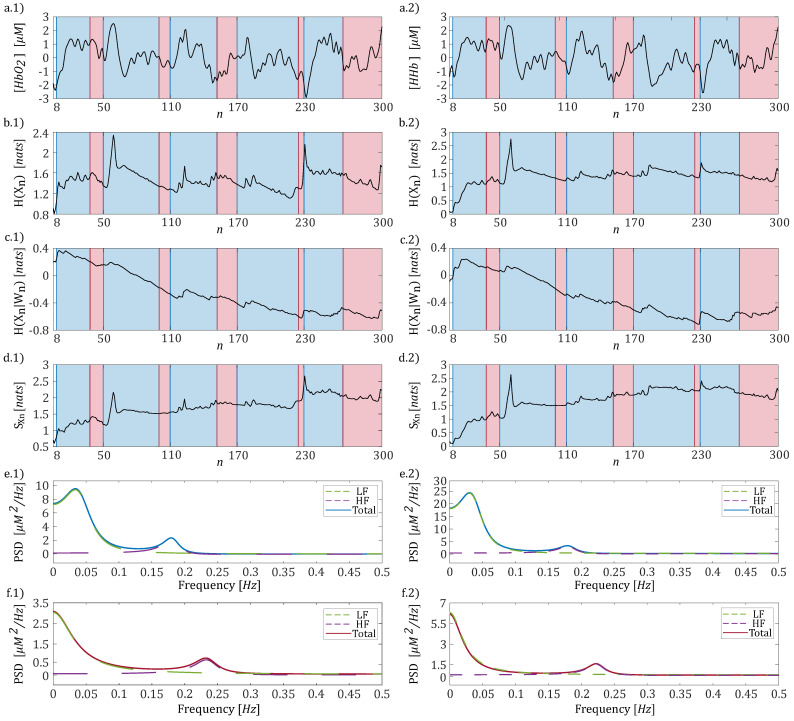
Time series showing the variation in hemoglobin concentration over a period of 5 min (panels (**a.1,a.2**)) obtained for a representative channel for $HbO_{2}$ (panel (**a.1**–**f.1**)) and HHb (panel (**a.2**–**f.2**)). The blue and red windows correspond to the time intervals associated with the apnea (blue area) and breathing (red area) phases, respectively. Time-resolved analysis of entropy (panels (**b.1**,**b.2**)), conditional entropy (panels (**c.1**,**c.2**)), and information storage (panels (**d.1**,**d.2**)) obtained for $HbO_{2}$ and HHb, respectively. PSD trends along with their spectral decompositions obtained for the apnea (panels (**e.1**,**e.2**)) and breathing phases (panels (**f.1**,**f.2**)) computed separately for $HbO_{2}$ and HHb, respectively.

The pre-processing pipeline employed in this study included the following steps: (i) the raw intensity signals were converted to optical density (OD) signals using the equation $OD=-\ln(I\left(t\right)/I_{o})$, where I(t) is the time-dependent recorded signal intensity and $I_{o}$ is its initial value [4]; (ii) the Temporal Derivative Distribution Repair (TDDR) method was applied to the OD signals as implemented in the fNIRS Brain AnalyzIR Python toolbox [31]. TDDR is a motion correction algorithm designed to eliminate the two most common motion artifacts in fNIRS data: spikes and baseline shifts; (iii) the corrected OD signals were converted to hemoglobin concentration signals ($HbO_{2}$ and HHb) using the modified Beer–Lambert law [32], following the procedure described in [4]. The resulting signals were then low-pass-filtered with a cutoff frequency of 0.5 Hz to prevent aliasing and subsequently downsampled to 1 Hz; (iv) finally, a high-pass autoregressive (AR) filter with infinite impulse response (IIR) and zero phase [33], with a cutoff frequency of 0.018 Hz, was applied to emphasize variations in the respiratory frequency band induced by the task. The resulting time series, each consisting of N=300 samples, were normalized to zero mean and unit variance. Representative trends for one channel and one subject are shown in Figure 2a.1 for $HbO_{2}$ and Figure 2a.2 for HHb, highlighting the variations observed during the breath phases (red windows) and the apnea phases (blue windows).

### 2.2. Time-Resolved Information Measures

Information-theoretic measures provide powerful approaches to assess the regularity and predictability of the dynamics of a given system. While traditional analyses provide overall measures of complexity and predictability, recent advancements extend these measures to enable time-resolved characterization [24,34,35]. Here, we apply these methods to study the transient evolution of scalp hemodynamics during breath-holding tasks—revealing dynamic responses in extracerebral oxygenation that conventional fNIRS analyses cannot resolve.

The analysis of any given dynamical system can be performed by mapping the system activity with a set of random variables and then studying the statistical dependencies among the observed realizations of the variables collected in the form of time series. In the general field of information theory, starting from the basic concept of entropy introduced by Shannon [36], it is possible to dissect the information processed in a dynamical system into meaningful elements of computation to quantify the complexity and predictability of the aforementioned system.

The $HbO_{2}$ or HHb signal, acquired for each subject and channel, was modeled as a realization of a zero-mean stochastic process *X*, with $X_{n}$ representing the random variable sampling the process at time *n* (temporal counter). Then, the Shannon entropy (H) can be used to quantify the amount of information needed to describe $X_{n}$ as follows: $H\left(X_{n}\right)=-E\left[\log p\left(x_{n}\right)\right]$, where $x_{n}$ refers to a realization of $X_{n}$ and $p(x_{n})$ is the probability density function of $X_{n}$ measured for the outcome $x_{n}$, and E[·] is the expectation operator computing the statistical average over all possible values of $x_{n}$. Assuming *X* as a Markov process, its past history can be truncated up to a lag *p* to obtain the p-dimensional vector $W_{n}={\left[X_{n-1},\ldots ,X_{n-p}\right]}^{⊤}$. To account for the dynamical evolution of the system *X*, it is possible to quantify the new information carried by the present state $X_{n}$ of the process which cannot be inferred from its past $W_{n}$, i.e., the Conditional Entropy (CE) [37]: $H\left(X_{n}|W_{n}\right)=-E\left[\log p\left(x_{n}|w_{n}\right)\right]$ where $w_{n}$ refers to a realization of $W_{n}$ and p(·|·) is the conditional probability. Specifically, the higher the CE, the more complex the dynamics generated by the system. Entropy and CE serve as measures to introduce the Information Storage (IS), which quantifies the amount of information contained in the present state that can be predicted by the knowledge of its past state [38] and can be defined as

(1) $$S_{X,n}=I\left(X_{n};W_{n}\right)=E\left[\log\frac{p(x_{n}|w_{n})}{p(x_{n})}\right].$$

The IS measures the predictability of the process at time *n* by quantifying the average level of uncertainty about the current state of the process $X_{n}$ that can be resolved by knowledge of its past states $W_{n}$.

To describe the time-resolved behavior of the fNIRS signal ($HbO_{2}$, HHb) during breathing and apnea phases, a time-varying analysis of the measures of H, CE, and IS was performed, through the adoption of a time-varying version of an autoregressive (TV-AR) model [24,34]:

(2) $$X_{n}=\sum_{k=1}^{p}a_{k,n}X_{n-k}+U_{n},$$

where $U_{n}$ represents the prediction error, while $a_{k,n}$ denotes the AR coefficient describing the interaction from $X_{n-k}$ to $X_{n}$ at lag *k*, relevant to the time instant *n*. Then, under Gaussian assumption of $X_{n}$, the time-resolved H can be expressed as follows [24]: $H\left(X_{n}\right)=\frac{1}{2}log\left(2\pi e\cdot \sigma_{X_{n}}^{2}\right)$, where $\sigma_{X_{n}}^{2}$ represents the variance of $X_{n}$. Moreover, if $X_{n}$ and $W_{n}$ are jointly Gaussian, the time-resolved CE of $X_{n}$ given $W_{n}$ can be expressed as follows [39]: $H\left(X_{n}|W_{n}\right)=\frac{1}{2}log\left(2\pi e\cdot \sigma_{U_{n}}^{2}\right)$, where $\sigma_{U_{n}}^{2}$ is the variance of the prediction error $U_{n}$ at the time step *n*. Thus, the equation given in (1) can be rewritten as

(3) $$S_{X,n}=H\left(X_{n}\right)-H\left(X_{n}|W_{n}\right)=\frac{1}{2}log\frac{\sigma_{X_{n}}^{2}}{\sigma_{U_{n}}^{2}}.$$

The identification procedure of the TV-AR model (2) can be performed through the recursive least-squares (RLS) as described in [40]. Briefly, the RLS consists of the following steps: (i) choose a value for the adaption factor c∈(0,1) and an order *p* of the AR model; (ii) define proper initial conditions for the vector of coefficients at time *p*, $A_{p}=\left[a_{1,p},...,a_{p,p}\right]\in R^{1\times p}$ and for the correlation matrix of the past state of *X* stored in $W_{n}$,$\Sigma_{W_{n}}=E\left[W_{n}W_{n}^{⊤}\right]\in R^{p\times p}$; (iii) considering *N-p* consecutive time steps, for n=p+1 to *N*, repeat the following steps:

(4a)ΣWn=(1−c)ΣWn−1+WnWn⊤,(4b)Kn=(ΣWn)−1Wn,(4c)Zn=Xn−An−1Wn,(4d)An=An−1+ZnKn⊤,

where $K_{n}\in R^{p\times 1}$ is the so-called gain vector and $Z_{n}\in R^{1\times 1}$ is intended as the a priori estimation error before updating the AR coefficients vector. The parameter (1 − *c*) controls the memory of the algorithm allowing it to follow possible statistical variations in the property of *X* in non-stationary conditions. To complete the identification procedure, a recursive estimation of the time-varying innovation variance can be obtained as follows: $\sigma_{U_{n}}^{2}=\sigma_{U_{n-1}}^{2}+c\left(Z_{n}^{2}-\sigma_{U_{n-1}}^{2}\right)$ [24,34]. The required recursive estimation of the process variance $\sigma_{X_{n}}^{2}$ can be directly derived from the structure of the linear TV-AR representation of the process *X* as described in [24].

### 2.3. Spectral Analysis of fNIRS Signals

Biomedical signals are often rich with oscillatory content, and therefore naturally lend themselves to spectral representation. Classical approaches integrating the power spectral density profile within the spectral bands of interest attempt to obtain band-specific time-domain powers, while the method of spectral decomposition used here allows for focusing only on the spectral components with frequencies within those bands, thus avoiding spurious contributions due to broadband oscillations [41].

To characterize the oscillatory content of each fNIRS signal ($HbO_{2}$, HHb), a linear model governed by the following difference equation was employed:

(5) $$X_{n}=\sum_{k=1}^{p}a_{k}X_{n-k}+U_{n},$$

where $U_{n}$ is the prediction error with a variance $\sigma_{U}^{2}$ and $a_{k}$ is the AR coefficient describing the interaction from $X_{n-k}$ to $X_{n}$ at lag *k*. The linear model (5) can be represented in the Z-domain through its Z-transform yielding X(z)=H(z)U(z), where $H\left(z\right)={\left[1-\sum_{k=1}^{p}a_{k}z^{-k}\right]}^{-1}$ is the transfer function relating the Fourier Transform (FT) of *U* to the FT of the process *X*. Applying the residue theorem, H(z) can be expressed as follows [41]:

(6) $$H\left(z\right)=\frac{z^{p}}{\prod_{k=1}^{p}\left(z-p_{k}\right)}=\prod_{k=1}^{p}H^{\left(k\right)}\left(z\right),$$

where $p_{k}$, k=1,…,p, are the *p* poles of the AR process, while the terms $H^{\left(k\right)}\left(z\right)=\frac{z}{z-p_{k}}\cdot \frac{1/z^{*}}{1/z^{*}-p_{k}}$ are pole-specific factors associated each with a given pole $p_{k}$, with * indicating the Hermitian transpose. The power spectral density (PSD) of the process *X* can be expressed in the Z-domain as $P\left(z\right)=H\left(z\right)\sigma_{U}^{2}H^{*}\left(\frac{1}{z^{*}}\right)$. Then, by using the Heaviside decomposition, it can be expanded into simple fractions corresponding to the poles of the system. These include the poles inside the unit circle, $p_{k}$, and their reciprocals outside the unit circle, ${\bar{p}}_{k}=p_{k}^{-1}$, for k=1,…,p, weighted by the residuals of P(z), specifically $r_{k}p_{k}$ and $-r_{k}p_{k}^{-1}$. This results in the following expression [41]:

(7) $$P\left(z\right)=\sum_{k=1}^{p}P^{\left(k\right)}\left(z\right)=\sum_{k=1}^{p}[\frac{r_{k}p_{k}}{z-p_{k}}-\frac{r_{k}p_{k}^{-1}}{z-p_{k}^{-1}}],$$

where the residuals are given by $r_{k}=\frac{\sigma_{U}^{2}}{z\prod_{h\neq k}\left(z-p_{h}\right)\cdot \prod \left(z^{-1}-p_{h}\right)}{|}_{z=p_{k}}$, k=1,…,p. Evaluating P(z) on the unit circle of the complex plane, specifically ${P\left(f\right)=P\left(z\right)|}_{z=e^{j2\pi f/f_{s}}}$, where $f\in [-f_{s}/2,f_{s}/2]$ and $f_{s}$ is the sampling frequency (1 Hz), allows for the derivation of the spectral profile, P(f), along with its $k^{th}$ component, $P^{\left(k\right)}\left(f\right)$. Each spectral component is characterized by a unique profile, which is determined by the central frequency of the oscillation, derived from the argument of the pole ($f_{k}=\frac{\arg(p_{k})}{2\pi }$), and by the power related to the residual of the pole. For real poles, the power is $\sigma_{k}^{2}=r_{k}$, and for complex conjugate poles, it is $\sigma_{k}^{2}=r_{k}+r_{k}^{*}$. Notably, the total variance $\sigma_{X}^{2}$ of the process is equal to the sum of the variances of all poles, $\sigma_{k}^{2}$, for k=1,…,p.

The computation of the PSD of *X* relies on the identification procedure of the AR model (5) which herein was performed through the ordinary least-squares (OLS) method [42]. By recalling the *p*-dimensional vector $W_{n}$ containing the past states of *X* and considering N consecutive time steps, a compact representation of the VAR model (5) can be defined as X=AW+U, where $A=[a_{1},\ldots ,a_{p}]$ is the 1×p vector of unknown coefficients, $X=[X_{p+1},\ldots ,X_{N}]$ and $U=[U_{p+1},\ldots ,U_{N}]$ are 1×(N−p) vectors collecting the present states and the residuals, and $W=[W_{p+1},\ldots ,W_{N}]$ is a p×(N−p) matrix collecting the regressor terms. Then by using the OLS formula an estimate of the AR coefficient can be obtained as follows: $\hat{A}=X{\left(W\right)}^{⊤}{\left[W{\left(W\right)}^{⊤}\right]}^{-1}$. The innovation process can be estimated as the residual time-series $\hat{U}=X-\hat{A}W$, whose ${\hat{\sigma }}_{U}^{2}$ is an estimate of the innovation variance.

### 2.4. Data Analysis and Statistical Validation

All the analyses were performed by considering separately the concentrations of $HbO_{2}$ and HHb for each channel and for each subject. Regarding their spectral analysis, two distinct identification procedures for the model (5) were performed, separately analyzing the breathing phase and the apnea task. Specifically, given $O=\left[O_{1},\ldots ,O_{6}\right]=\left[8,50,110,170,230,300\right]$ as a vector representing the time instants of the apnea task onsets, with the last element indicating the total length of the time series, we defined $l_{i}$ and $m_{i}$, i∈{1,…,5}, as the i-th time window during which the subjects breathed normally (red windows in Figure 2) or performed the apnea task, respectively (blue windows in Figure 2). Let $B_{i}$ denote a generic instant when the subject begins to breathe normally after an apnea task (which may differ for each apnea event and subject). Using this notation, we can define the following time windows: $m_{i}=\left[O_{i}+\left(p+1\right),\ldots ,B_{i}-1\right]$, and $l_{i}=\left[B_{i}+\left(p+1\right),\ldots ,O_{i+1}-1\right]$. Then when the breath task was considered, we used $X=[X_{l_{1}},\ldots ,X_{l_{5}}]$ as the present state, while the matrix of regressors W was defined by simply switching the content of each window $l_{i}$ of one temporal unit in the past, up to *p* lags. The same rationale was used for the apnea task where $X=[X_{m_{1}},\ldots ,X_{m_{5}}]$. This procedure was implemented to prevent potential discontinuities that could arise from simply concatenating different time windows and to increase the number of observations, thereby reducing the estimation bias [43]. Spectral profiles were computed using (7), and the low-frequency (LF) and high-frequency (HF) components were identified based on poles with central frequencies in the ranges [0.04–0.15] Hz and [0.15–0.4] Hz [44]. The PSD profile relevant to each pole, $P^{\left(k\right)}\left(f\right)$, was then integrated within the whole frequency spectrum to obtain the power content associated with the LF and HF components, respectively. A representative example of the spectral decomposition performed on one representative channel for $HbO_{2}$ and HHb is reported in Figure 2 (panel e: apnea; panel f: breath). The code used for performing the Power Spectral Density Analysis can be found here: https://github.com/YuriAntonacci/LSP_toolbox/tree/main, accessed on 12 December 2024. To test whether the spectral content of fNIRS signals was modulated during the breath-holding task, a statistical comparison between the distributions of central frequency and spectral power obtained across subjects during the breath and apnea phases was performed using the paired non-parametric Wilcoxon signed-rank test (α<0.05).

As for the time-resolved analysis of the different information-theoretic measures, the identification procedure of the TV-AR model (2) was performed setting p=4 and (1−c)=0.975. The model order *p* was selected using the Akaike Information Criterion (AIC) [42] as a guide. Analysis across the entire scalp and for all subjects returned values ranging from four to six. To ensure consistency and avoid duplicate or negative peaks in the subsequent spectral analysis, the model order was fixed at four [44]. As regard for the forgetting factor (1−c), the selection was performed according with previous studies, which highlighted the interval [0.97, 0.98] as an ideal compromise for the bias–variance trade-off [24,34].

The time-resolved measures of entropy ($H(X_{n})$), conditional entropy ($H(X_{n}|W_{n})$), and information storage ($S_{X,n}$) (panels b–d of Figure 2) were then computed and averaged within each *i*-th time window corresponding to the analyzed experimental condition (breath and apnea). The obtained values were further averaged across the five available windows for each experimental condition to obtain a representative value for each of the six subjects. Statistically significant differences between the apnea and breath conditions were evaluated for each information-theoretic measure using the Wilcoxon test for paired data (α<0.05). The code necessary to compute time-resolved information measures can be found here: https://github.com/YuriAntonacci/Time-VaryingIS, accessed on 12 December 2024.

Therefore, a measure of the effect size was also determined to assess the magnitude of the differences observed among experimental conditions. Specifically, denoting with $\mu_{S_{1}}$, $\mu_{S_{2}}$, $\sigma_{S_{1}}^{2}$, and $\sigma_{S_{2}}^{2}$, the mean and the variance of two distributions $S_{1}$ and $S_{2}$ obtained measuring the mean and the SD of the distribution across subjects of each information theoretic measure, we computed the Cohen’s d measure defined for equally sized groups as follows [45]:

(8) $$d=\frac{\mu_{S_{1}}-\mu_{S_{2}}}{\sqrt{(\sigma_{S_{1}}^{2}+\sigma_{S_{2}}^{2})/2}}.$$

Typically, a small effect size arises for d=0.2, a medium effect size between 0.2 and 0.8, and large was considered when d=0.8 [45]. Finally, we assessed the statistical power of each test to evaluate how the sample size (N = 6) would influence the results. Statistical power (1−β) represents the probability of correctly rejecting a false null hypothesis (i.e., the likelihood of detecting a true physiological effect if one exists).

## 3. Results

### 3.1. Frequency Specific Analysis of fNIRS Signals

Panels e,f of Figure 2 present the spectral decomposition of the PSD for $HbO_{2}$ (panel 1) and HHb (panel 2) signals from one representative subject during the apnea (panel e) and breath (panel f) phases. Regardless of the analyzed time series ($HbO_{2}$ or HHb), the PSD trends reveal two distinct peaks centered at approximately 0.05 Hz and 0.2 Hz with the latter showing a modulation when the respiratory activity is considered.

**Figure 3:**
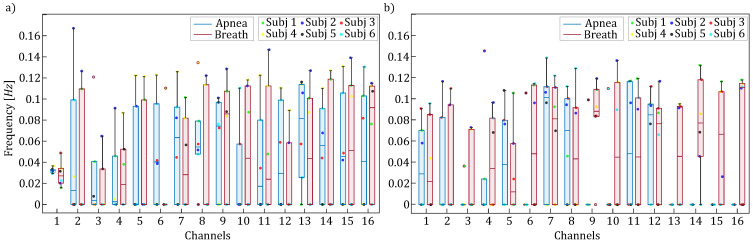
Boxplot distributions and individual values of the frequency locations of the LF spectral peaks for $HbO_{2}$ (panel (**a**)) and HHb (panel (**b**)) are shown for each channel, separately for apnea (blue) and breath (red) phases.

**Figure 4:**
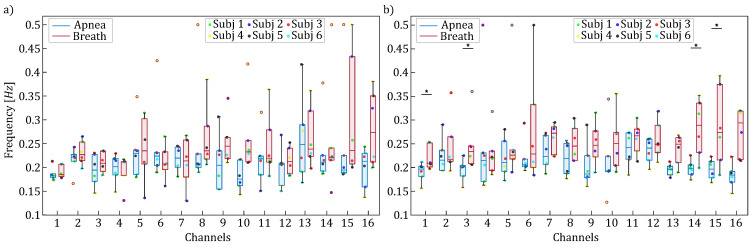
Boxplot distributions and individual values of the frequency locations of the HF spectral peaks for $HbO_{2}$ (panel (**a**)) and HHb (panel (**b**)) are shown for each channel, separately for apnea (blue) and breath (red) phases. Statistically significant differences between breath and apnea windows are indicated with * (*p* < 0.05, Wilcoxon signed rank test).

Figure 3 and Figure 4 report the boxplot distributions illustrating the frequency locations of the LF and HF spectral peaks for the apnea (blue) and breath (red) phases, computed separately for $HbO_{2}$ (panel a) and HHb (panel b). Figure 3 displays the distribution of spectral peak frequencies within the [0.04–0.15] Hz frequency band, which reveals negligible modulation between apnea and breathing phases, indicating that respiratory activity has limited influence on low-frequency oscillations.

Figure 4 displays the distribution of spectral peak frequencies within the [0.15–0.4] Hz frequency band, revealing significant task-dependent shifts. Overall, regardless of the time series analyzed (i.e., $HbO_{2}$ or HHb), there is a consistent trend of increased oscillation frequency observed during the breathing task across all fNIRS channels. This increase becomes statistically significant for HHb, occurring in four specific channels (Figure 4b). Remarkably, the corresponding Cohen’s *d* values consistently exceed 1.2 (1−β>0.6), indicating a strong difference between the two experimental conditions.

**Figure 5:**
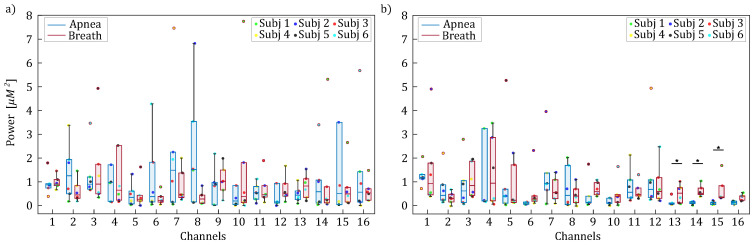
Boxplot distributions and individual values of the spectral power averaged over the LF ([0.04–0.15] Hz) frequency band for $HbO_{2}$ (panel (**a**)) and HHb (panel (**b**)), computed for each channel and displayed separately for the apnea (blue) and breath (red) phases. Statistically significant differences between breath and apnea windows are marked with * (*p* < 0.05, Wilcoxon signed rank test).

**Figure 6:**
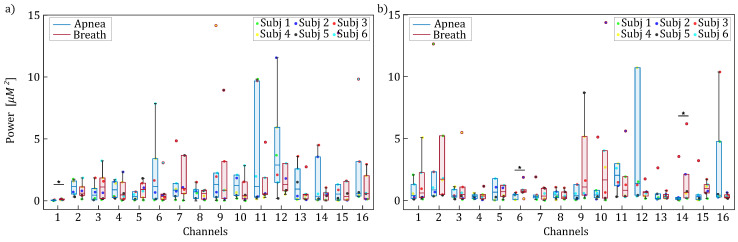
Boxplot distributions and individual values of the spectral power averaged over the HF ([0.15–0.4] Hz) frequency band for $HbO_{2}$ (panel (**a**)) and HHb (panel (**b**)), computed for each channel and displayed separately for the apnea (blue) and breath (red) phases. Statistically significant differences between breath and apnea windows are marked with * (*p* < 0.05, Wilcoxon signed rank test).

Figure 5 and Figure 6 display the boxplot distributions obtained for the spectral power of the fNIRS signal relevant to the LF and HF frequency bands, respectively. The results highlight an increase in both LF and HF power associated with breathing activity. Though this increase is statistically significant only for a few channels, when HHb is considered, the corresponding values of Cohen’s *d* are always greater than 1 (1−β>0.55), highlighting the presence of a strong modulation of the spectral content of fNIRS signals as an effect of respiratory activity.

### 3.2. Time-Resolved Information-Theoretic Measures

**Figure 7:**
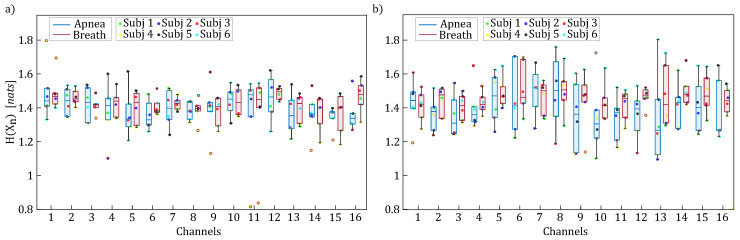
Boxplot distributions and individual values of the time-resolved entropy ($H(X_{n})$) for $HbO_{2}$ (panel (**a**)) and HHb (panel (**b**)), computed for each channel and displayed separately for the apnea (blue) and breath (red) phases.

**Figure 8:**
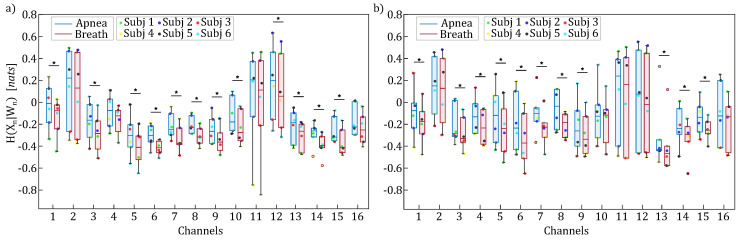
Boxplot distributions and individual values of the time-resolved conditional entropy ($H(X_{n}|W_{n})$) for $HbO_{2}$ (panel (**a**)) and HHb (panel (**b**)), computed for each channel and displayed separately for the apnea (blue) and breath (red) phases. Statistically significant differences between the breath and apnea windows are marked with * (*p* < 0.05, Wilcoxon signed-rank test).

**Figure 9:**
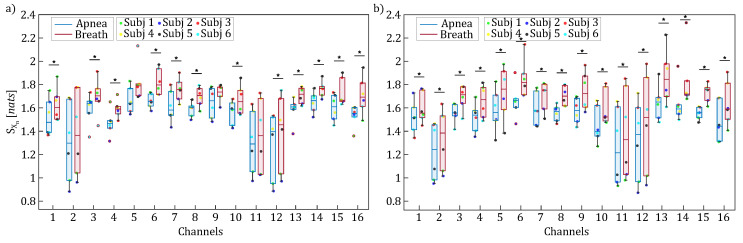
Boxplot distributions and individual values of the time-resolved information storage ($S_{X,n}$) for $HbO_{2}$ (panel (**a**)) and HHb (panel (**b**)), computed for each channel and displayed separately for the apnea (blue) and breath (red) phases. Statistically significant differences between the breath and apnea windows are marked with * (*p* < 0.05, Wilcoxon signed-rank test).

Figure 7, Figure 8 and Figure 9 display the boxplot distributions of the time-resolved information measures of entropy, conditional entropy, and information storage, averaged across five different time windows relevant to the apnea (blue color) and breath (red color) phases, computed separately for $HbO_{2}$ (panel a) and HHb (panel b). The results in Figure 7 suggest a modulation of uncertainty in the oxyhemoglobin and deoxyhemoglobin time series during breathing compared to the apnea condition. However, this difference is not statistically significant, indicating that the amount of information contained in the concentrations of oxyhemoglobin and deoxyhemoglobin remained largely unchanged despite the breath-holding task. Conversely, Figure 8 and Figure 9 reveal statistically significant modulations of the time-resolved conditional entropy and the information storage measures, respectively. In detail, the analysis of time-resolved conditional entropy reveals a statistically significant decrease in the complexity of scalp hemodynamics during breathing periods, regardless of the hemoglobin time series considered, and across nearly all analyzed channels. In contrast, time-resolved information storage shows a statistically significant increase in the predictability of scalp hemodynamics during the same periods. A variability can be observed in these measures across different channels even though Figure 8 and Figure 9 consistently show Cohen’s d values > 0.6 with statistical power (1−β)>0.8.

## 4. Discussion

The main results of this work can be summarized as follows: (a) the presence of two different peaks in the PSD of fNIRS signals centered approximately at 0.05 Hz and 0.2 Hz and mainly related to vascular and respiratory components [46,47,48,49]; (b) the modulation in both the amplitude and frequency of oscillations in the time series of oxyhemoglobin and deoxyhemoglobin concentrations during the breath-holding task, which may be sustained by the strong coupling between blood pressure and the hemodynamic responses measured in extracerebral tissues [22,50]; (c) the presence of a transition in the information processing characteristics of fNIRS dynamics associated with the shift between breathing and apnea phases, which is marked by an increase in signal predictability during respiratory activity—an effect that cannot be captured using simple measures such as Shannon entropy.

The results reported in Figure 3 and Figure 5 highlighted the presence of a vascular component, known as Mayer waves, consisting of rhythmic fluctuations in arterial pressure caused by changes in vasomotor tone, typically occurring within frequencies ≤0.1 Hz, which are reflected in the hemodynamic oscillations of superficial scalp tissues, thereby modulating the optical fNIRS signals [47]. The reduced amplitude of low-frequency components (<0.1 Hz) observed during apnea, even if limited to a few channels, may be attributed to the induction of hypercapnia, i.e., an increase in CO_2_ levels during breath-holding tasks in humans [51]. Indeed, the temporary suspension of breathing activity leads to a rise in carbon dioxide levels, triggering a vasodilation response. This vasodilation increases cerebral blood flow, which directly modulates the amplitude of low-frequency oscillations associated with Mayer waves [52].

The results displayed in Figure 4 and Figure 6 revealed the presence of a spectral component in the 0.15–0.4 Hz range, which can be attributed to respiratory activity. This component reflects respiratory-driven variations in arterial pressure as well as in cerebral and extracerebral blood flow. The observed shift in frequency location, occurring exclusively within the HF band, has been previously reported and is physiologically linked to increased respiratory rates during the breathing phase compared to the apnea phase [53]. Interestingly, the peak relevant to the HF range was also observed during apnea and reflects fundamental respiratory physiology: while voluntary breath-holding suppresses overt chest movements, it cannot interrupt the brainstem’s central respiratory rhythm [54]. We speculate that these HF oscillations can be related to respiratory sinus arrhythmia—a well-documented phenomenon where autonomic nervous system activity causes a person’s heart rate to oscillate in phase with the residual respiratory drive, even during apparent apnea [55]. However, the expected increase in the spectral content within HF components during the breathing phase, as reported in previous studies, was not clearly observed in our analysis—possibly due to the limited duration of the experimental period, which should ideally last at least 28–30 s to reliably capture such changes [56].

Overall, our results revealed dynamic changes in both vascular and respiratory components for oxygenated and deoxygenated hemoglobin, consistent with established mechanisms of respiratory–cardiovascular coupling [54]. Previous studies show that spontaneous hemodynamic oscillations, although not directly elicited by external stimuli or tasks, can still exhibit variations in amplitude and frequency. These fluctuations significantly affect frequency-domain analyses, as they shape the spectral profile of the recorded signals and underscore the influence of underlying physiological rhythms on hemodynamic activity [11,57,58,59].

The results shown in Figure 7, Figure 8 and Figure 9 indicated less-complex fNIRS signal dynamics associated with the presence of respiratory activity. Although not directly comparable with previous studies characterizing brain dynamics and physiological systems using information-theoretic approaches [24,34,38,60], these works consistently show that the emergence of a predominant oscillation—often driven by synchronization phenomena—is associated with increased predictability of brain dynamics across different contexts [61]. These findings are consistent with the PSD analysis of fNIRS signals dynamics herein performed, where the breathing task was associated with an increase in the PSD within both the LF and HF bands. Moreover, since a transition occurs after the onset of apnea, we can link the observed decrease in predictability to previous studies, which have highlighted that such transitions are associated with a reduction of the time-resolved information storage [34]. Lastly, we observed a variability in the information-theoretic measures across fNIRS channels, which may originate from the following: (i) spatial heterogeneity in fNIRS sensitivity (superficial vs. deep tissue contributions) [14]; (ii) regional variations in neurovascular coupling [62]; and (iii) task-specific activation patterns [63]. Despite this channel-wise variability, the measures of time-resolved information storage and conditional entropy consistently showed Cohen’s d values > 0.6 with statistical power (1−β)>0.8, indicating robust effects of breath-holding on both time-resolved conditional entropy and information storage.

### Final Remarks and Limitations

Our results demonstrated that integrating time-resolved information-theoretic measures (entropy, conditional entropy, information storage) with parametric spectral analysis can yield novel insights into fNIRS signal dynamics. Power spectral density analysis revealed distinct spectral signatures: Mayer waves (0.04–0.15 Hz) exhibited minimal modulation during breath-holding, suggesting autonomic stability, while high-frequency oscillations (0.15–0.4 Hz) persisted during apnea, reflecting the ongoing influence of the central respiratory rhythm and respiratory sinus arrhythmia. Time-resolved measures further showed that the presence of respiratory activity reduces hemodynamic complexity and increases signal predictability compared to apnea, underscoring the role of respiration in shaping scalp-measured hemodynamic activity.

The main limitation of our study is that the presented results cannot be directly attributed to cortical activity, as CW single-distance NIRS is known to be more sensitive to superficial tissues than to brain tissue. The measured signals likely reflect a superposition of responses from multiple depths, with a substantial contribution from scalp hemodynamics [14]. Indeed, both incident and backscattered light traverses highly vascularized extracerebral layers such as the skin, subcutaneous fat, and scalp muscles, making the signals particularly sensitive to hemodynamic fluctuations in these regions [30].

Finally, the generally low statistical power and small effect sizes observed in our analyses suggest that some nonsignificant results may be attributed to insufficient statistical sensitivity, primarily due to the limited sample size. This represents a key limitation of the study and highlights the need for larger datasets to enable more robust statistical assessments of the observed effects.

## 5. Conclusions

This study highlights the significance of fNIRS monitoring and advanced signal processing techniques in exploring scalp hemodynamics during physiological tasks, specifically the breath-holding task. As a non-invasive and portable modality, fNIRS offers a unique capability to monitor real-time hemodynamic responses, complementing other neuroimaging methods [4]. Our findings support the value of a multi-domain analytical approach for reinterpreting physiological “noise” as evidence of functional interactions within the framework of network physiology, and demonstrate the feasibility of applying advanced signal processing techniques even with prototype CW fNIRS systems. Indeed, combining fNIRS with information-theoretic measures allows for the identification of subtle dynamics in both cerebral and extracerebral functions, thereby deepening the physiological insights that can be gained from fNIRS data.

Our results reveal that novel aspects of respiratory–scalp coupling could enable practical applications in the following: (i) clinical monitoring of patients with impaired respiratory–cerebral coupling, such as those with sleep apnea or cerebrovascular disorders; (ii) passive brain–computer interfaces, where entropy-based measures could provide real-time assessment of cognitive state [64,65]. Future work should focus on the validation of these measures in larger cohorts (N > 30 subjects), comparing performance across different fNIRS systems (including time-domain devices), and developing hybrid fNIRS-EEG protocols to better separate neural and vascular contributions.

## Data Availability

The data presented in this study are available on request from the corresponding author.
